# Editorial: III Bio.Natural-bioactive natural products research meeting: pharmacology perspectives

**DOI:** 10.3389/fphar.2024.1517673

**Published:** 2024-11-19

**Authors:** Patricia Rijo, Lillian Barros, Thomas Efferth

**Affiliations:** ^1^ CBIOS—Lusófona University’s Research Center for Biosciences and Health Technologies, Lisbon, Portugal; ^2^ Instituto de Investigação do Medicamento (iMed.ULisboa), Faculdade de Farmácia, Universidade de Lisboa, Lisbon, Portugal; ^3^ CIMO, LA SusTEC, Instituto Politécnico de Bragança, Campus de Santa Apolónia, Bragança, Portugal; ^4^ Department of Pharmaceutical Biology, Institute of Pharmacy and Biochemistry, Johannes Gutenberg University, Mainz, Germany

**Keywords:** natural products (NP), Bio.Natural meeting, pharmacology, ethnopharmacology, natural bioactives

This Research Topic arises from the III Bio.Natural - Bioactive Natural Products Research Meeting, held on July 13–14, 2023, a forum dedicated to exploring the vast potential of natural products. Building on the success of the first two editions in 2019 and 2021 in Lisbon, this third edition continues to emphasize the significant contributions of natural products research, particularly their pharmacological properties and therapeutic applications.

Hosted by Frontiers in Pharmacology, this Research Topic aims to highlight the critical value of natural products, with a special focus on their pharmacological aspects. This Research Topic underscores the unique and enduring role that natural products play in modern pharmacology, showcasing contributions from leading researchers in the field. It features four original research works and three review articles, each exploring different dimensions of natural products and their potential for therapeutic applications.

We are excited to bring together researchers from around the world to share their latest findings and contribute to this ever-evolving field. The III Bio.Natural Meeting, along with this accompanying Research Topic, celebrates the diversity and potential of natural products in shaping the future of pharmacology. Below are key studies featured in this Research Topic:

Sarwar et al. investigated the ethanol extract of Panicum antidotale, revealing its significant anti-inflammatory, wound-healing, analgesic, and antipyretic properties, supported by antioxidant activity and molecular docking studies.

Sun et al. reviewed the role of mitochondrial dysfunction in eye disorders such as glaucoma, age-related macular degeneration (AMD), and diabetic retinopathy (DR), while highlighting the therapeutic potential of natural products in targeting these dysfunctions.

Abdeen et al. explored the protective effects of *Chlorella vulgaris* (ChV) in mitigating aflatoxin-induced nephrotoxicity and its impact on egg quality, offering insights into potential strategies for counteracting aflatoxin-associated toxicity in humans and animals.

Hassan et al. examined the potential protective effects of whey protein isolate (WP) against gamma irradiation-induced lingual damage, highlighting its bioactivity and ability to mitigate radiation-induced harm to surrounding tissues in oral cancer treatments.

Yao et al. explored the modulation of the Vitamin D receptor (VDR) by traditional Chinese medicines (TCMs) and bioactive compounds, highlighting their potential therapeutic applications in VDR-dependent diseases. This review provides a comprehensive overview of VDR’s genetic expression, function, and structure, while discussing the mechanisms behind TCMs and bioactive compounds that target this receptor.

Laddha and Kulkarni investigated the protective effects of Daidzein, a soy isoflavone, in alleviating diabetic peripheral neuropathy in Sprague Dawley rats. The study shows that Daidzein treatment significantly reduced plasma glucose levels, improved sensory functions, and alleviated symptoms such as mechanical hyperalgesia and allodynia. It also highlights Daidzein’s potential to improve nerve conduction velocities and inhibit oxidative stress through NOX-4 modulation, suggesting its therapeutic potential for diabetic neuropathy.

Chauhan et al. reviewed the potential of Ursolic Acid (UA) in preventing gastrointestinal cancer by modulating key signaling pathways involved in cancer development. UA’s anti-inflammatory, anti-proliferative, and anti-metastatic properties make it promising, although its clinical use is limited by low oral bioavailability and poor permeability. The review emphasizes the use of nanoformulations, like liposomes and polymer micelles, to enhance UA’s stability and bioavailability, highlighting its future prospects in GI cancer treatment.

In conclusion, this Research Topic serves as a testament to the invaluable role of natural products in advancing modern pharmacology. The contributions featured here, spanning original research and review articles, underscore the multifaceted potential of natural products for therapeutic applications. As we continue to uncover the pharmacological properties of these compounds, their enduring importance in drug discovery and development becomes ever more apparent. The work presented in this Research Topic not only deepens our understanding of the molecular mechanisms of natural products but also provides new insights into their clinical potential, reinforcing the significance of continued exploration in this dynamic and promising field. The III Bio.Natural Meeting and this accompanying Research Topic embody the bright future of natural products research.

